# Decoding the prenatal clock of sheep muscle fiber type differentiation: a temporal map from embryonic to mature types

**DOI:** 10.3389/fcell.2025.1649640

**Published:** 2025-09-01

**Authors:** Zhenzhen Gu, Weiwei Duan, Chenxi Liu, Wenrong Li, Ning Zhang, Bin Han, Mingjun Liu

**Affiliations:** ^1^ College of Life Science and Technology, Xinjiang University, Urumqi, China; ^2^ Key Laboratory of Genetics Breeding and Reproduction of Grass Feeding Livestock, Ministry of Agriculture and Rural Affairs, Xinjiang Academy of Animal Science, Urumqi, China; ^3^ Key Laboratory of Animal Biotechnology of Xinjiang, Xinjiang Academy of Animal Science, Urumqi, China; ^4^ Institute of Animal Biotechnology, Xinjiang Academy of Animal Science, Urumqi, China

**Keywords:** sheep, longissimus thoracis et lumborum, fiber type, myosin heavy chain, RNA-seq

## Abstract

**Background:**

The composition of skeletal muscle fiber types is a crucial determinant of meat quality in livestock. While the transition from embryonic to mature fiber types is known to occur during late gestation, the precise temporal dynamics and molecular mechanisms underlying this process in sheep remain poorly understood. A comprehensive, multi-technique approach is needed to elucidate the critical developmental transitions in myofiber specification.

**Methods:**

We systematically investigated muscle fiber type differentiation in the longissimus thoracis et lumborum (LTL) muscle of sheep fetuses at 85, 105, 115, and 135 days of gestation (D85-D135) using an integrated approach combining histological (ATPase staining), protein biochemical (SDS-PAGE and Western blotting), and transcriptomic (RNA-seq) analyses. This multi-omics strategy enabled comprehensive characterization of fiber type composition, myosin heavy chain (MHC) isoform expression, and associated molecular pathways.

**Results:**

Our findings revealed distinct stage-specific developmental patterns. Prior to D105, the number of muscle fibres increased progressively, with fibres predominantly expressing embryonic (MHC-emb) and neonatal (MHC-neo) isoforms. After D105, fibre numbers stabilized and underwent maturation, transitioning to predominantly type I, IIA, and IIB fibres, with type IIA fibres becoming the most abundant (61.2%) by D135. Transcriptome analysis identified D105 as a critical transition point, characterized by the significant downregulation of MYH3 (MHC-emb) and MYH8 (MHC-neo), and the upregulation of mature fibre genes (MYH7, MYH2, and MYH4). Concurrently, we observed increased expression of oxidative metabolism genes (COX7A1, NDUFB6) and enhanced aerobic metabolic capacity in maturing fibers.

**Discussion:**

This study provides the first integrated multi-omics characterization of muscle fiber type development in late-gestation sheep, identifying D105 as a pivotal transition point in myofiber specification. Our findings reveal coordinated molecular and metabolic changes underlying the transition from embryonic to mature fibre types, with significant implications for understanding ruminant muscle development. These results establish a scientific foundation for improving meat quality through targeted molecular breeding strategies and prenatal nutritional interventions in sheep production systems.

## Introduction

Skeletal muscle fibre type composition is a critical determinant of muscle function, influencing metabolic efficiency, contraction properties, and overall meat quality in livestock animals. During development, mammalian skeletal muscle undergoes a dynamic transition from immature fiber types (embryonic and neonatal) to mature fibers (types I, IIA, and IIB/IIX), each exhibiting distinct metabolic and contractile characteristics. Type I fibers are slow-twitch and oxidative, while type IIA fibers are fast-twitch and oxidative, and type IIB/IIX fibers are fast-twitch and glycolytic (Schiaffino and Reggiani, 2011). This transition is particularly important in livestock species where muscle characteristics directly influence meat quality and production efficiency (Picard and Gagaoua, 2020; Park et al., 2022; Costa et al., 2021). Recent advances in developmental biology have revealed that this process is regulated by an intricate interplay of intrinsic genetic programs and extrinsic environmental factors, including maternal nutrition and hormonal signals (Listrat et al., 2016; Zhou et al., 2021; Polizel et al., 2021).

The molecular basis of muscle fiber differentiation centers on the regulated expression of myosin heavy chain (MHC) isoforms, which serve as both structural components and functional markers of fiber types (Hoh, 2023). Developmental isoforms such as MHC-emb (MYH3) and MHC-neo (MYH8) are transiently expressed during early stages, while mature isoforms like MHC-I (MYH7), MHC-IIa (MYH2), and MHC-IIb (MYH4) dominate in later stages. The transition from immature to mature fiber types is accompanied by significant shifts in energy metabolism, with oxidative pathways becoming increasingly prominent as the fibers mature. Understanding these developmental trajectories is essential for elucidating the mechanisms underlying muscle growth and adaptation, as well as for improving meat quality through targeted breeding and nutritional interventions.

Currently, researchers can accurately classify muscle fiber types by: 1) measuring myosin heavy chain (MHC) protein expression, 2) quantifying myosin heavy chain genes (MYHs) mRNA levels, and 3) assessing metabolic characteristics of the fibers (Blemker et al., 1985; Sawano and Mizunoya, 2022; Lefaucheur, 2010). However, existing studies predominantly focus on single techniques (such as histology or transcriptomics), lacking integrative multi-omics analysis. This limits a comprehensive understanding of fiber type differentiation. Integrative multi-omics analyses are needed to bridge the gap between morphological, protein, and gene expression data. Moreover, while the prenatal development of muscle fibers has been explored in species such as cattle and pigs, research in sheep remains limited, particularly regarding the temporal dynamics of fiber type transitions during late gestation (da Costa et al., 2003; Albrecht et al., 2013; Wang et al., 2024).

This study aims to decode the prenatal clock of muscle fiber type differentiation in sheep by integrating ATPase staining, SDS-PAGE, Western blotting, and RNA sequencing (RNA-seq) to characterize the LTL muscle at key gestational stages (D85, D105, D115, and D135). By identifying critical time points and molecular signatures of fiber type transitions, we aim to gain insights for molecular breeding strategies and prenatal nutritional management, as well as support agricultural efforts to enhance meat quality in sheep.

## Materials and methods

### Animals

The sheep used in this study were bred from a research flock of Chinese Merino sheep raised at the sheep breeding base of the Biotechnology Research Center of Xinjiang Academy of Animal Sciences. Adult Chinese Merino sheep of the aged 2.5 ± 0.5 years, in good physical condition, and weighing 47.3 ± 2.1 kg were selected for our study. Following oestrus synchronization treatment, intrauterine artificial insemination was performed laparoscopically via semen from a single Chinese Merino ram to ensure genetic consistency across all experimental subjects. The ewes were group-housed (4 animals per 48 m^2^ pen) in a semi-confined system with free access to an outdoor paddock (80 m^2^). Each pen featured a covered resting area with rubber matting and automatic waterers (10 °C–15 °C). Animals were fed *ad libitum* with: 1) forage (60% of diet; mixed alfalfa-oat hay: 14.2% CP, 42.3% NDF), and 2) concentrate (40%; barley-corn-soybean meal-mineral mix) formulated to meet NY/T 816-2004 standards. Fresh feed was provided twice daily with weekly intake monitoring. The ewes were killed by a captive bolt pistol and exsanguinated at 85, 105, and 135 days of gestation. The fetuses were removed via cesarean section and killed via cardiac puncture with sodium pentobarbitone. *LTL* tissue was collected from a singleton fetus on days (D) 85 (three males), D105 (two males and one female), D115 (two males and one female), and D135 (three females and one male) of gestation. At the same time, collect LTL tissue from 3-month-old lambs (one female). The samples were divided into two parts. One batch was placed in 4% paraformaldehyde, and the other was stored in liquid nitrogen.

### H&E staining

*LTL* tissues were fixed in phosphate-buffered 4% paraformaldehyde, embedded in paraffin, and sliced into 5 μm sections. The slices were immersed in xylene for 30 min and then treated with 100% ethanol for 3 min. Then, they were treated with gradient ethanol solutions of 95%, 90%, 80%, and 70% for 1 min. Next, the slices were stained with hematoxylin for 10 min and eosin for 8 min, and the muscle structure was observed under an optical microscope (Olympus, BX51TRF, Japan).

### ATPase staining

The reagents and experimental methods used in this study are described by Guth and Samaha (1970). Briefly, serial transverse sections (8-µm thick) were prepared using a cryostat at −20 °C and then dried for 30 min at room temperature. Sections were stained for actomyosin ATPase after preincubation in acidic and alkaline solutions at pH 4.8 and pH 10.7, respectively, for 10 min.

### Myosin extraction

Approximately 20 mg of *LTL* was homogenized in 400 µL of extraction buffer composed of 0.5 M sodium chloride, 20 mM sodium pyrophosphate, 50 mM Tris, 1 mM EDTA, and 1 mM dithiothreitol. The homogenate was then incubated on ice for 10 min and centrifuged at 2,500 × g for 10 min at 4 °C. The total protein concentration in each supernatant was determined via a Pierce™ BCA Protein Assay Kit (Thermo, 23227) after the samples were adjusted to equal concentrations. The extracted proteins were mixed with an equal volume of loading buffer composed of 4% (w/v) SDS, 125 mM Tris (pH 6.8), 17% (v/v) glycerol (100%), 10% (v/v) β-mercaptoethanol, and 0.02% (w/v) pyronin Y, incubated at room temperature for 10 min and heated at 100 °C for 10 min before separation by SDS-PAGE.

### Myosin separation by gradient SDS-PAGE

The MHC isoforms were separated via SDS-PAGE, as described by Hemmings et al. (Hemmings et al., 2009). The samples were run in duplicate on 1.0-mm-thick gels by using the Protean II system (Bio-Rad, United States). The concentrations of the stacking and resolving gels were 6% and 9%, respectively. Notably, both gels also contained 47% (v/v) and 34% (v/v) glycerol (100%). The upper running buffer consisted of 100 mM Tris, 150 mM glycine, 0.1% (w/v) SDS, and 10 mM β-mercaptoethanol. The lower running buffer consisted of 50 mM Tris, 75 mM glycine, and 0.05% (w/v) SDS. All these reagents were prepared with distilled water. Electrophoresis was performed at 0 °C and a constant voltage of 200 V for 50 h. Afterward, the gels were stained overnight with 0.2% Coomassie blue R250 and then destained in a mixture of 30% ethanol (v/v) and 5% acetic acid (v/v).

### Western blotting

The samples and gel concentrations used for Western blotting were the same as those used for the electrophoretic separation of MHC isoforms, except that a 0.75-mm-thick gel and 1 µg of protein per lane were used for Western blotting. The samples were run at a constant voltage of 70 V for 33 h at 4 °C using the Mini-PROTEAN Tetra system (Bio-Rad, United States) and then transferred onto polyvinylidene fluoride membranes. The membranes were then incubated in a blocking solution (Prepared by dissolving 5 g of skim milk powder in 100 mL of TBST buffer solution) for 2 h at room temperature to block nonspecific antibody binding. The membranes were subsequently incubated overnight at 4 °C with anti-MHC-fast (dilution 1:1000; abcam, ab91506), MHC-slow (dilution 1:1000; abcam, ab11083), and anti-actin (dilution 1:2000; Abmart, T40001) antibodies. After being washed four times for 5 min in TBST, the membranes were incubated with a secondary HRP-conjugated solution. Goat anti-rabbit IgG (H + L) (dilution 1:1000, Beyotime A0208) or HRP-conjugated goat anti-mouse IgG (H + L) (dilution 1:2000; Beyotime, A0216) was used for 1 h. Antibody signals were detected with an ECL Chemiluminescent Substrates Kit (Biosharp BL520A, China) and visualized with a GE AI600 imaging instrument (GE AI600, United States). The Western blotting experiment was repeated three times.

### RNA library construction and RNA sequencing (RNA-seq)

Total RNA was extracted from the *LTL* of Chinese Merino sheep fetuses at gestational stages D85, D105, or D135 via TRIzol reagent (Life Technologies, United States). The RNA quality and content were assessed via an Agilent 2100 Bioanalyzer (Agilent Technologies, United States) and a NanoDrop ND-2000 Spectrophotometer (NanoDrop Technologies, United States), respectively. Additionally, agarose gel electrophoresis was employed to assess RNA integrity and purity. For mRNA sequencing, RNA samples were first treated with RNase R to generate fragments of 250–300 bp. Complementary DNA (cDNA) was synthesized using random oligonucleotide primers, DNA polymerase I, and dNTPs, followed by end repair, A-tailing, and ligation of sequencing adapters. The resulting cDNA was then amplified by PCR to construct the sequencing library. The Illumina HiSeq 4000 sequencing platform from Novogene Bioinformatics Technology Co., Ltd. (Beijing, China) was used for double-terminal (pair-end) high-throughput sequencing.

### RNA-seq quality assessment and gene expression analysis

Raw sequencing reads were processed using Fastp software (Chen et al., 2018) to remove adapters, poly-N sequences, and low-quality reads, yielding clean reads for subsequent analyses. Each sample generated more than 10 Gb of clean data. The quality of the sequencing data was evaluated using parameters such as the Q20, Q30, GC content, and sequence duplication levels with FastQC software. The Q30 value was >88%, and the GC base content was 50%. All downstream analyses were performed on high-quality data. Clean reads were mapped to the sheep reference genome (http://ftp.ensembl.org/pub/release-104/gtf/ovis_aries_rambouillet/) via HISAT2 software (BKim et al., 2015). Over 90% of our samples’ clean reads were mapped to the ovine reference genome (Supplementary Table S1). Transcript assembly was performed using StringTie software (Pertea et al., 2015), and mRNA levels were quantitatively analyzed with StringTie-eB software (Bray et al., 2016). Gene expression levels were estimated via the fragments per kilobase of transcript per million fragments (FPKM) method.

### RT-qPCR

Total RNA (*LTL* tissues) was extracted via TRIzol reagent and then reverse transcribed via the Fast King RT Kit (TIANGEN, KR116, China) following the manufacturer’s instructions. Following the manufacturer’s protocol, we performed RT-qPCR using primers (Supplementary Table S2) and Fast SYBR™ Green Master Mix (Roche, 4913914001, Switzerland) to quantify the expression levels of MYH3, MYH8, MYH1, MYH7, MYH2, and MYH4 at different gestational stages. The RT-qPCR experiment was repeated three times, and the relative abundance of each gene was determined via the 2^−ΔΔCt^ method (Livak and Schmittgen, 2001).

### Principal component analysis

Principal component analysis (PCA) was performed via R statistical software (version 3.0.3) with the ggplot2 package (version 2.2.1) for visualization.

### Differential gene expression analysis

EdgeR software was used to identify differentially expressed genes (DEGs) between different developmental stages (Smyth and McCarthy, 2010). Differential gene expression was based on a |log_2_ (fold change)| ≥1 and adjusted *P*-value (FDR) < 0.05.

### Gene ontology (GO) and Kyoto encyclopedia of genes and genomes (KEGG) pathway enrichment analyses

ClusterProfiler (R package) was used for GO (http://geneontology.org) and KEGG (http://www.genome.ad.jp/kegg/) functional enrichment analysis of the data. The display of relevant annotation results was performed via the OmicStudio tool at https://www.omicstudio.cn/tool. The GO terms and KEGG pathways significantly associated with the DEGs were determined on the basis of the corresponding *P*-values. GO terms or KEGG pathways with a *P*-value <0.05 were considered significantly enriched. Functional evidence was obtained on the basis of the relationships between significant GO terms or pathways and the genes.

### Analysis of differentially expressed gene trends

This analysis was performed using Short Time-series Expression Miner (STEM) software (Ernst and Bar-Joseph, 2006). First, the differentially expressed gene matrix screened between groups underwent log2 normalization and was organized into a time series according to the experimental time points (D85, D105, D135). For the analysis, we preset 8 potential expression change profiles and employed a correlation coefficient-based K-means clustering algorithm for trend classification, with trends showing P-value <0.05 considered statistically significant.

### Statistics

The results of ATPase staining were manually counted under an optical microscope (OLYMPUS, BX51TRF, Japan). The total number of fibers counted for each gestational stage exceeded 1,000. The percentage of each fiber type was calculated by dividing its count by the total number of fibers. The data are presented as the means ± SEMs. The target protein bands from the Western blot analysis were quantified via ImageJ software, with β-actin serving as the internal loading control. Statistical analyses were performed using one-way ANOVA, considering gestational day as the fixed effect and fetus as the random effect. Significance levels were set at *P < 0.05, **P < 0.01, and ***P < 0.001; nonsignificant results (P > 0.05) are denoted as “ns”. Relative gene expression levels were determined using the comparative 2^−ΔΔCT^ method, with 18S rRNA as the endogenous reference and the D85 group as the calibrator control.

## Results

### Identification of muscle fiber types on the basis of ATPase activity

**FIGURE 1:**
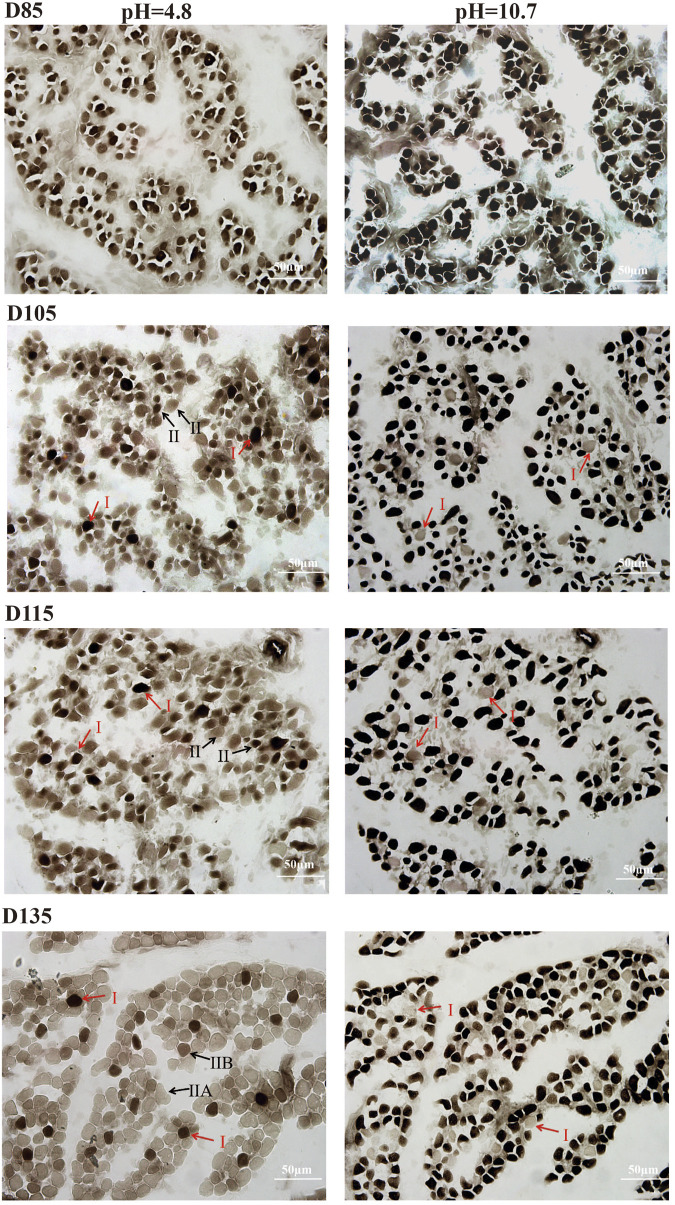
ATPase activity of fetal *LTL* in D85-D135. Type I fibers stain black, type IIB fibers stain brown, and type IIA fibers stain light brown. Scale bar = 50 µm.

This study systematically investigated the developmental dynamics of muscle fibers in fetal sheep during late gestation, integrating structural and functional analyses to elucidate stage-specific maturation patterns. Histological observations revealed that by D85, primary muscle fibers and nascent muscle bundle structures had formed, while by D105, the fibers exhibited a highly organized parallel arrangement with peripheral nuclei, indicating advanced structural maturation. Notably, post-D105, muscle fiber hyperplasia ceased, and growth shifted to hypertrophy, with fibers increasing significantly in length and diameter (Supplementary Figure S1). These structural changes were closely associated with the functional differentiation of fiber types, as demonstrated by ATPase staining. Prior to D105, no distinct fiber types could be identified, but coinciding with the structural maturation observed in Supplementary Figure S1, type I fibers became discernible by D105–D115. However, type IIA and IIB fibers remained indistinguishable until D135, when all three fiber types (I, IIA, IIB) were clearly differentiated (Figure 1), corresponding to the phase of rapid hypertrophic growth. At this stage, type I, IIA, and IIB fibers accounted for 8.39%, 61.22%, and 30.39% of the total population, respectively (Table 1), reflecting the establishment of functional diversity concurrent with structural maturation.

**TABLE 1:** The number and percentage of myofiber types at different gestation stages.

pH=4.8 gestation	Type І	Type ІІA	Type ІІB	Type І %	Type ІІA%	Type ІІB %
D105	20 ± 2.40	241 ± 15.86		7.52%	92.48%	
D115	21 ± 1.41	232 ± 6.68		8.30%	91.70%	
D135	22 ± 2.59	162 ± 33.41	80 ± 16.12	8.39%	61.22%	30.39%

*Note:* Fiber types ⅡA and ⅡB at D105 and D115 are indistinguishable from each other.

### Identification of muscle fiber types on the basis of MHC isoforms

**FIGURE 2:**
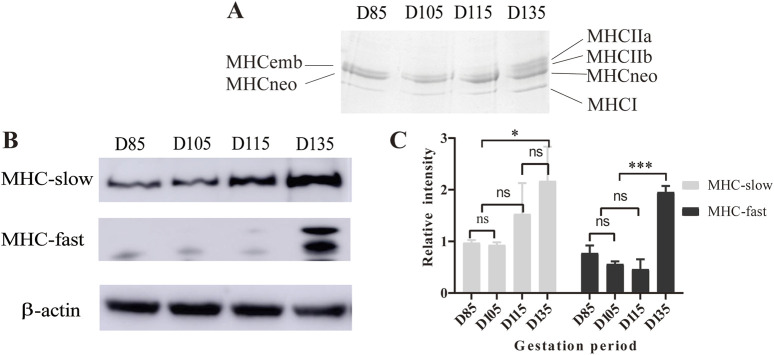
Analysis of MHC isoforms by SDS-PAGE and Western blotting. **(A)** Electrophoresis results with 12 µg of sample in each lane. **(B)** Western blot of MHC isoforms of *LTL* from D85 to D135. **(C)** Statistical results of the Western blot gray values. *P < 0.05, ***P < 0.0005, ns *P* > 0.05.

To confirm the timing of the appearance of type IIA and IIB fibers and the presence of embryonic (emb) and neonatal (neo) fiber types, this study used SDS-PAGE to analyze the composition of MHC subtypes in the fetal erector spinae muscle during the D85-D135 period. Gel electrophoresis revealed five MHC bands related to migration speed, namely, MHC-IIa, MHC-IIb, MHC-emb, MHC-neo, and MHC-I. When the loading amount is 12 μg, MHC-embs and MHC-neo can be separated (Figure 2A). To accurately determine the positions of MHC-IIa and MHC-IIb, we also analyzed the composition of MHC isoforms in the *LTL* tissue of Chinese Merino sheep at 3 months of postnatal age (Supplementary Figure S2). The results revealed three MHC isoforms, namely, MHC-emb, MHC-neo, and MHC-I, at D85 and D105. As the fetus developed, the levels of MHC-emb and MHC-neo gradually decreased and eventually disappeared at D135 and postnatally, respectively. In contrast, the isoforms of MHC-IIa and MHC-IIb became detectable from D135 onward.

To further evaluate the MHC composition and expression during sheep development, we performed Western blotting with antibodies against fast and slow MHC isoforms. The Western blotting results revealed that the expression of fast-MHC isoforms (MHC-Ⅱa and MHC-Ⅱb) was extremely low and undetectable at D85-115. However, they were abundant on D135. On the other hand, the slow-MHC isoform (MHC-І) was present throughout the period from D85 to D135, but its expression gradually increased from the early to the late fetal stage (Figures 2B,C). The Western blot results and gradient SDS-PAGE results both indicate that MHC-IIa and IIb isoforms can be detected at D135, which is consistent with the results of ATPase staining.

### Identification of muscle fiber types on the basis of genes

A muscle fiber classification method based on the expression profile of muscle fiber type marker genes is one of the most widely accepted classification methods currently available. Because previous histological analysis revealed that the composition of muscle fiber D105 is similar to that of D115, we performed transcriptome sequencing of fetuses at D85, D105, and D135.

**FIGURE 3:**
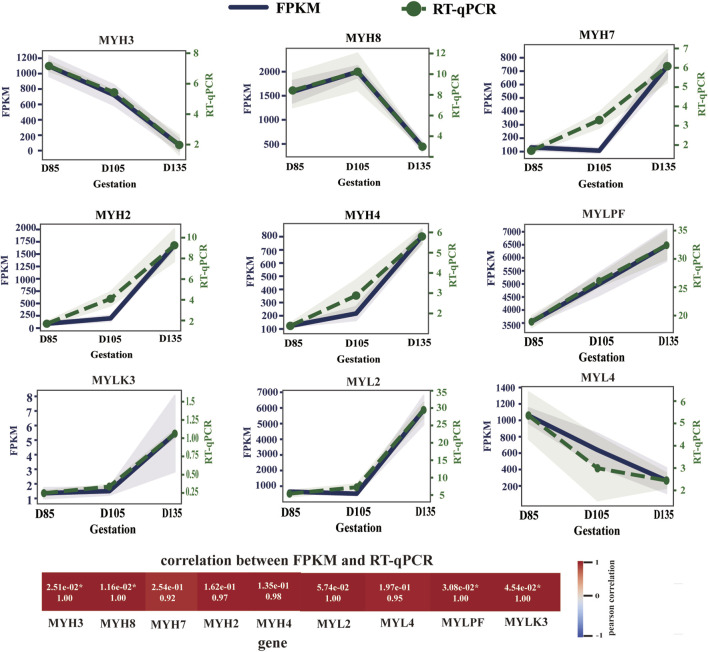
Expression trends of myosin heavy chain and myosin light chain genes. The level of gene expression at each time point is expressed as the mean ± SEM.

Transcriptome data analysis revealed that the key time point for fetal muscle fiber type development is D105. Before D105, high expression of MYH3 and MYH8 was observed, while the expression of mature muscle fiber genes (MYH7, MYH2, and MYH4) remained very low. However, after D105, the expression patterns of muscle fibre-type marker genes underwent significant changes. The expression of MYH3 and MYH8 decreased sharply, whereas the expression of MYH7, MYH2 and MYH4 increased rapidly. By D135, the muscle fiber composition had changed to a mature type, characterized by the highest expression of MYH2, followed by MYH7 and MYH4, whereas MYH8 remained only at very low levels, and MYH3 was almost undetectable (Figure 3). This dynamic change clearly indicates that the transition of muscle fibers from embryonic and neonatal types to mature types I, IIA, and IIB occurs primarily after D105, culminating in the formation of mature muscle tissue dominated by type IIA muscle fibers.

We also observed the expression of MLC2s (MYL2 gene) in slow myofibers, MLC2f (MYLPF gene) in fast myofibers, MLC1a/emb (MYL4 gene) in developing skeletal muscle, and MYLK3, the enzyme responsible for catalyzing MYL2 phosphorylation, at the transcriptional level (Figure 3). During fetal development, MYL4 expression decreased, whereas MYL2, MYLPF, and MYLK3 expression continued to increase, particularly after D105.

### Transcriptome profile reveals the metabolic characteristics of fetal skeletal muscle

**FIGURE 4:**
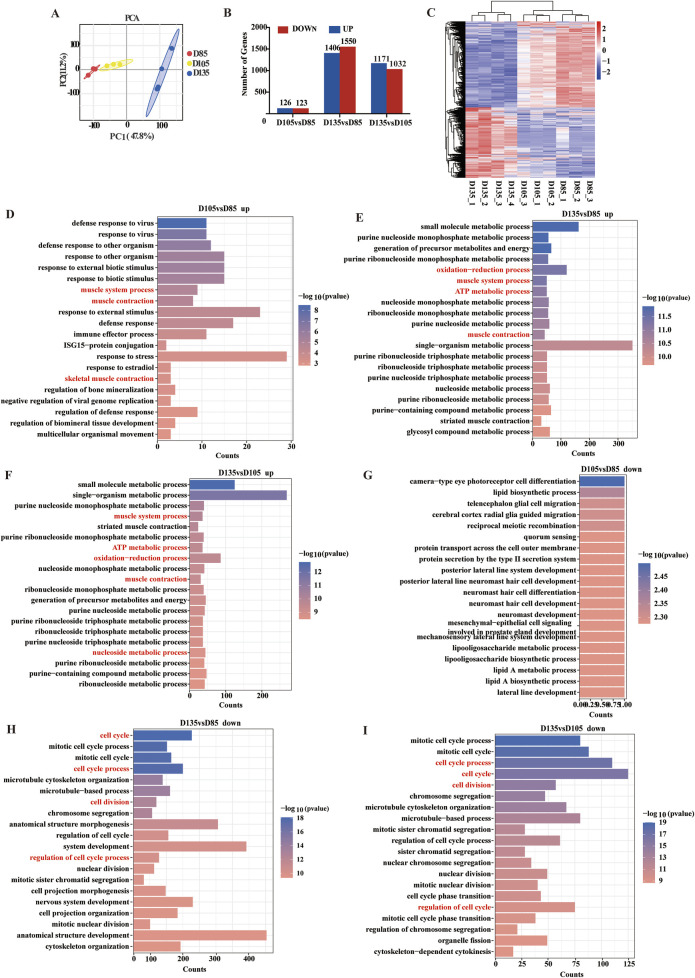
Differential genes and functional analysis. **(A)** PCA analysis of mRNA. **(B)** Statistics of DEGs. **(C)** Heatmap of DEGs. **(D–F)** The top 20 biological processes (BP) terms enriched with the upregulated genes. **(G–I)** The top 20 BP terms enriched with the downregulated genes.

The energy metabolism characteristics of muscle depend mainly on its muscle fiber type composition. To explore the metabolic characteristics of fetal skeletal muscle and further clarify the composition of muscle fiber types, RNA-Seq data were analyzed in depth. Principal component analysis revealed strong correlations between samples (Figure 4A). RNA-seq identified 12880 mRNAs with FPKM ≥1. Three pairwise comparisons were then performed between the gestational time points: D105 vs. D85, D135 vs. D85, and D135 vs. D105. The 3147 DEGs were identified through differential expression analysis, comprising 1510 upregulated genes and 1637 downregulated genes (Figure 4B). Cluster analysis of DEGs revealed distinct shifts in gene expression profiles throughout fetal development (Figure 4C). GO functional enrichment analysis of the DEGs revealed that the upregulated genes were enriched in oxidation-reduction processes, carbon metabolism, muscle system processes, muscle contraction, and striated muscle contraction. The downregulated genes were enriched in terms of cell division, the cell cycle, and the regulation of the cell cycle (Figures 4D–I).

**FIGURE 5:**
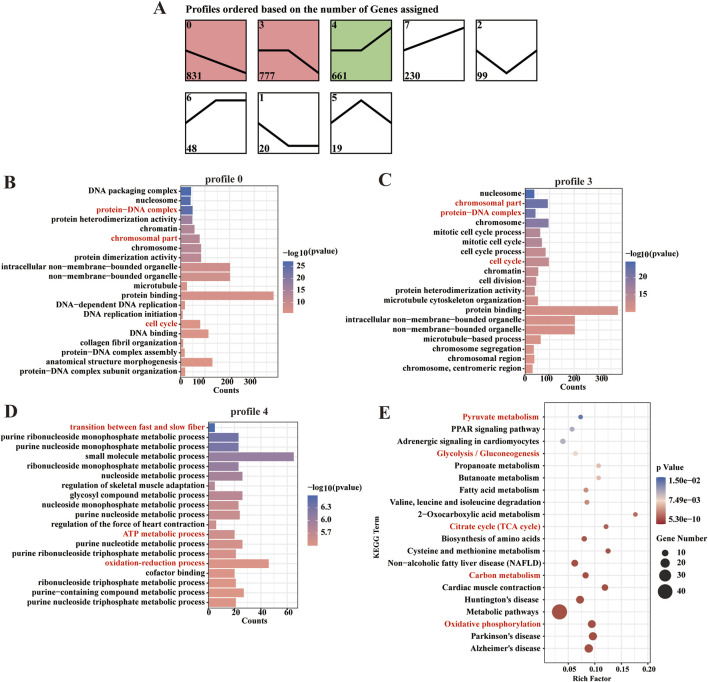
Patterns of expression and functional enrichment analysis of the DEGs. **(A)** Analysis of DEG expression patterns. **(B–D)** The top 20 GO terms in each combination. **(E)** Scatter plot of the top 20 pathways in profile 4.

To systematically characterize the dynamic changes in gene expression associated with energy metabolism during fetal muscle development, we performed a comprehensive time-series analysis of DEGs using the STEM software. This approach allowed us to identify distinct transcriptional trajectories and correlate them with critical phases of muscle fiber maturation. Eight candidate expression profiles were identified, of which three (profile 0, 3, and 4) exhibited statistically significant temporal trends (P < 0.05). Profile 0 contained 831 genes and profile 3 comprised 777 genes. Most notably, profile 4 included 661 genes that displayed marked upregulation after D105 (Figure 5A).

GO and KEGG pathway analyses revealed distinct functional specializations among these profiles. Genes in Profiles 0 and 3 were predominantly associated with cell cycle regulation, chromosomal organization, and protein-DNA complex assembly, consistent with their roles in myoblast proliferation and early differentiation. In contrast, Profile 4 genes were uniquely enriched in oxidation-reduction processes, ATP metabolism, and muscle fiber-type switching (e.g., transition between fast and slow fiber). This profile’s late activation suggests a metabolic shift coinciding with the maturation of contractile and oxidative machinery in developing muscle (Figures 5B–D). KEGG pathway analysis further highlighted the central role of profile 4 in energy metabolism, with robust enrichment in carbon metabolism, oxidative phosphorylation, glycolysis/gluconeogenesis, and the TCA cycle (Figure 5E). These findings indicate that after D105, fetal sheep muscle undergoes a pronounced metabolic transition, with aerobic oxidation becoming the dominant energy source to meet the increasing demands of maturing muscle fibers.

**FIGURE 6:**
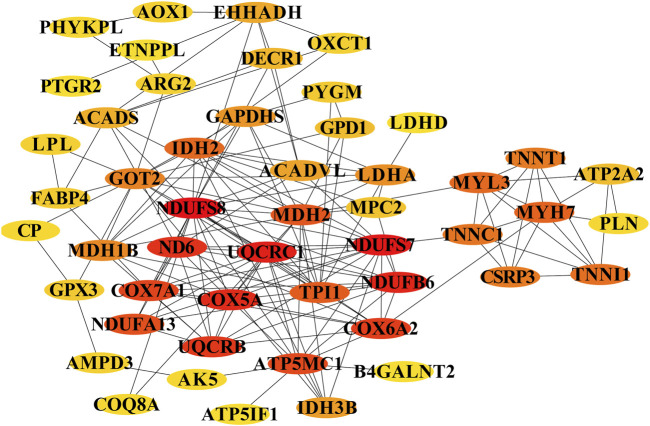
Core genes involved in energy metabolism in myofibers and type transition. The cycle nodes represent genes; the redder the color is, the more central the role within the network. The edges between two nodes represent interactions between genes.

To identify key regulators of muscle fiber development, we constructed a protein-protein interaction network from the top 50 hub genes in Profile 4 using Cytoscape. This analysis revealed several high-confidence candidates positioned at critical network nodes, including mitochondrial components (COX7A1, NDUFB6), calcium handling proteins (ATP2A2/SERCA2, TNNC1, TNNT1), and myosin heavy chain isoforms (MYH7). The centrality of these genes suggests they orchestrate the metabolic and structural development required for muscle function. For instance, the role of ATP2A2 in calcium reuptake may fine-tune the oxidative capacity of slow-twitch fibers, while MYH7 and troponin isoforms (TNNT1, TNNC1) could direct the assembly of fatigue-resistant fiber phenotypes. These results propose a two-phase model of fetal muscle development: an early phase (pre-D105) dominated by proliferative and structural programs, and a late phase (post-D105) marked by metabolic maturation and fiber-type specification (Figure 6).

## Discussion

With the rapid development of molecular biology techniques, classification methods for muscle fiber types have evolved from a morphological level to a molecular level. The establishment of protein analysis techniques such as ATPase staining, SDS-PAGE, and Western blot, as well as gene expression detection methods like immunohistochemistry and qRT-PCR, has provided multidimensional research tools for the precise identification of muscle fiber types. Owing to the dynamic changes in fiber composition and metabolic characteristics that occur during skeletal muscle development, a combination of multiple techniques is necessary to achieve accurate classification. In this study, through the integration of histological, proteomic, and transcriptomic analyses, we identified D105 as a key developmental milestone marking the transition from embryonic and neonatal fiber types to mature fibers (type I, IIA, and IIB). Notably, the expression profile of MHC proteins highly correlates with changes in MYH gene transcription levels, and the upregulation of oxidative metabolism genes synchronizes with the increase in the proportion of type IIA fibers. These findings reveal a synergistic regulatory relationship between muscle fiber type differentiation and metabolic trait development at the molecular mechanism level.

We used gradient SDS-PAGE to resolve the five MHC isoforms in fetal sheep for the first time. Our results demonstrated that the predominant protein isoforms transitioned from embryonic to neonatal and ultimately to adult types (I, IIA, and IIB). Previous studies have also shown that developmental myosin isoforms (MHC-emb and MHC-neo) are transitorily expressed during developmental stages but not in mature myofibers (Sartore et al., 1982; Agarwal et al., 2020). Maier et al. reported that in sheep, MHC-neo was undetectable between D140 of gestation and postnatal D28 (Maier et al., 1992). Adult-fast MHC types (MHC-ⅡA and MHC-ⅡB) have been detected during the fetal period in several species. For example, in cattle and sheep, the contraction and metabolic differentiation of myofibers occur primarily in the last third of gestation, with muscle expressing adult-fast MHC types at the end of gestation. In contrast, in pigs, expression starts after birth (Martyn et al., 2004). Furthermore, although MYH7 expression levels were consistently elevated in transcriptomic data, SDS-PAGE and Western blot results showed that MHC-I expression levels also gradually increased. However, based on ATPase results, the number of type I fibers did not increase; only their color gradually darkened. Previous studies have reported that changes in gene expression at the transcriptional and protein levels do not fully represent changes in the number of different types of muscle fibers; rather, they primarily reflect changes in muscle fiber metabolism and contractile properties (Hoppeler, 2016; Bottinelli and Reggiani, 2000; Pette and Staron, 2001).

The transition from embryonic (MHC-emb/MYH3) and neonatal (MHC-neo/MYH8) fibers to mature types (MHC-I, -IIa, and -IIb) after D105 aligns with the observed decline in MYH3/MYH8 expression and the upregulation of MYH7, MYH2, and MYH4. This shift coincides with a dramatic increase in oxidative metabolic capacity, as evidenced by the upregulation of genes such as COX7A1 (cytochrome c oxidase subunit) and NDUFB6 (NADH dehydrogenase ubiquinone flavoprotein), which are critical for mitochondrial electron transport and ATP synthesis, indicating that fiber type specification is tightly coupled with metabolic adaptation. The dominance of type IIA fibers (61.2%) at D135 further underscores this metabolic shift, as IIA fibers are oxidative-glycolytic hybrids with high mitochondrial density. This synergy ensures that mature fibers meet the energy demands of contraction through efficient aerobic pathways, thereby optimizing their performance. These findings suggest that D105 initiates not only structural maturation but also a metabolic transition from glycolytic reliance in immature fibers to aerobic oxidation in mature fibers—a process vital for postnatal muscle function and energy efficiency.

The STEM analysis clearly shows the coordinated upregulation of oxidative metabolism genes (including TCA cycle components IDH2, and fatty acid oxidation genes ACADVL) beginning at D105, coinciding with the observed shift toward oxidative type IIA fiber dominance (61.2% of fibers). Additionally, this study reinforces the connection between gene expression results and biological processes by elucidating enriched GO terms such as “muscle contraction” (represented by MYH7, MYH2, and TNNC1) and “oxidoreductase activity” (represented by COX7A1 and NDUFB6), as well as the KEGG “oxidative phosphorytation” pathway shared with oxidized skeletal muscle fibers. These transcriptional signatures collectively demonstrate a metabolic shift from glycolytic to oxidative states, which underlies the fiber type conversion observed in this study, providing compelling molecular evidence that complements and explains findings at the histological and protein levels regarding the timing and nature of muscle fibre maturation.

Studies have shown that coexpression rules govern the pairing of myosin light chain (MLC) isoforms with specific MHC isoforms, leading to a wide array of myosin isoforms (Schiaffino and Reggiani, 2011). Regulatory MLCs play crucial roles in modulating MHC activity. For example, phosphorylation of MYL2 enhances calcium sensitivity and augments the ATPase activity of MHC, thereby influencing muscle contraction (Morkin, 2000; Sweeney et al., 1993; Geeves et al., 2005; Vicente-Manzanares et al., 2009). At the same time, the phosphorylation modification of MYL2 prolongs the calcium ion binding time of slow muscle fibers (type I) and reduces the actin-MHC binding constant of fast muscle fibers (type II). This differentiated regulation lays the foundation for myofiber type specific differentiation. The expression patterns of MYL2, MYLPF, MYL4, and MYLK3 are consistent with the expression patterns of MYH genes in specific muscle fiber types. The increased expression of MYLK3 indicates a significant increase in MYL2 phosphorylation levels and MHC ATPase activity after D105, facilitating the formation of fast and slow myofiber types.

Sex is a significant factor influencing the development and transformation of muscle fiber types, and its mechanism of action may be closely related to the synergistic effects of hormones, genetics, and exercise (Fournier et al., 2022). The initial differentiation of muscle fiber types in sheep occurs during the fetal period, resulting in the formation of type I and type II muscle fibers. At this stage, the distribution of muscle fiber types has not yet exhibited significant sex differences (Prache et al., 2022). Fetal muscle development is regulated primarily by genetics, growth factors (such as IGF-1), and maternal environmental factors, whereas the effects of sex hormones become more pronounced postnatally (Li et al., 2021). Studies in cattle, sheep, and pig indicate that fetal-stage sex has a minimal effect on muscle fiber type, with primary differences emerging postnatally (especially during puberty) through the action of sex hormones (Li et al., 2021; Wicks et al., 2019; Greenwood et al., 2007).

## Conclusion

This study identified the critical time points (D105) for muscle fiber type development in sheep fetuses, as well as the temporal expression characteristics of embryonic, neonatal and mature (types I, IIA, and IIB) muscle fibers. Prior to D105, muscle fibers were in the formation stage, with their numbers showing a continuously increasing trend. At this stage, embryonic-type, neonatal-type, and type I muscle fibers coexist. After D105, muscle fiber development enters the maturation phase, during which the number of fibers stabilizes. The muscle fiber types transition to a composition pattern of neonatal type, type I, type IIA, and type IIB, with type IIA muscle fibers being the most dominant type, accounting for 61.2% of the total. The oxidative metabolic capacity of muscle fibers significantly increases after D105, and these fibers rely primarily on aerobic oxidation processes for energy supply.

## Data Availability

The datasets presented in this study can be found in online repositories. The names of the repository/repositories and accession number(s) can be found below: https://www.ncbi.nlm.nih.gov/ with accession no. PRJNA1083059.
