# Supplementary figures and images for: Decoding the prenatal clock of sheep muscle fiber type differentiation: a temporal map from embryonic to mature types
Source: Front Cell Dev Biol. 2025 Sep 1;13:1649640. doi: 10.3389/fcell.2025.1649640 (PMC12434085; doi:10.3389/fcell.2025.1649640)

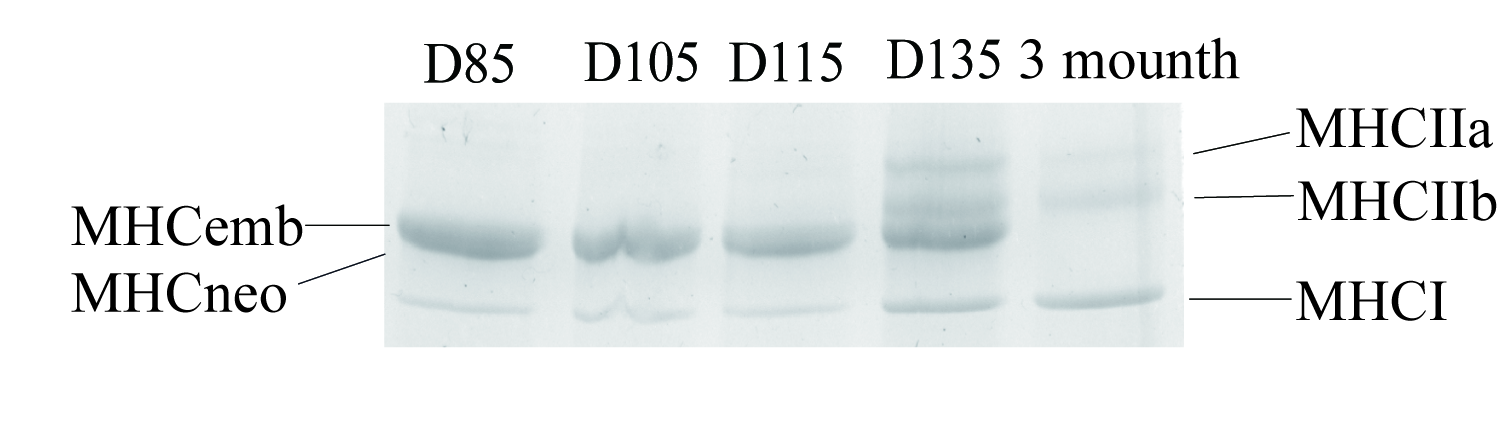

Supplement: Supplementary file 2 [file Image2.tif]

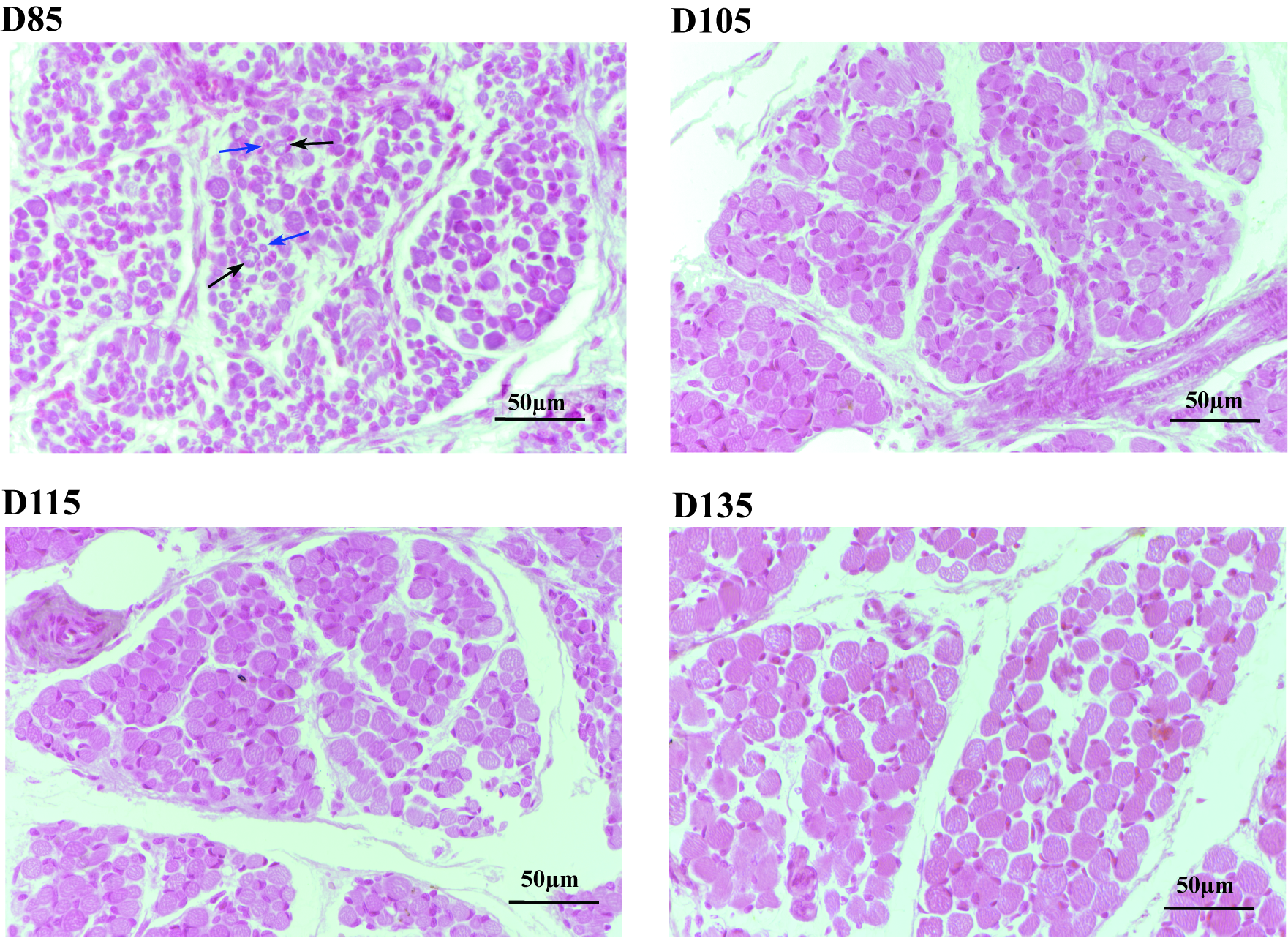

Supplement: Supplementary file 3 [file Image1.tif]
